# Impact of prediabetes on long-term cardiovascular outcomes in patients with myocardial infarction with nonobstructive coronary arteries

**DOI:** 10.1186/s13098-021-00721-9

**Published:** 2021-09-24

**Authors:** Side Gao, Wenjian Ma, Sizhuang Huang, Xuze Lin, Mengyue Yu

**Affiliations:** Department of Cardiology, Fuwai Hospital, National Center for Cardiovascular Diseases, Chinese Academy of Medical Sciences and Peking Union Medical College, Bei Li Shi Rd 167, Beijing, 100037 PR China

**Keywords:** Myocardial infarction with nonobstructive coronary arteries (MINOCA), Prediabetes, Cardiovascular outcomes

## Abstract

**Background:**

Abnormal glucose metabolism including diabetes (DM) and prediabetes (pre-DM) have been reported as predictors of poorer outcomes after acute myocardial infarction (AMI). However, the prognostic value of pre-DM in patients with myocardial infarction with nonobstructive coronary arteries (MINOCA) remains unclear.

**Methods:**

A total of 1179 MINOCA patients were prospectively recruited and divided into normoglycemia (NG), pre-DM, and DM groups according to glycated hemoglobin (HbA_1c_) levels or past history. The primary endpoint was a composite of major adverse cardiovascular events (MACE), including all-cause death, nonfatal MI, nonfatal stroke, revascularization and hospitalization for unstable angina or heart failure. Kaplan–Meier and Cox regression analyses were performed.

**Results:**

Patients with pre-DM and DM had a significantly higher incidence of MACE compared with NG group (10.8%, 16.1%, 19.4%; p = 0.003) over the median follow-up of 41.7 months. After multivariate adjustment, both pre-DM and DM were significantly associated with an increased risk of MACE (NG as reference; pre-DM: 1.45, 95% CI 1.03–2.09, p = 0.042; DM: HR 1.79, 95% CI 1.20–2.66, p = 0.005). At subgroup analysis, pre-DM remained a robust risk factor of MACE compared to NG. In addition, pre-DM had a similar impact as DM on long-term prognosis in patients with MINOCA.

**Conclusions:**

Pre-DM defined as raised HbA_1c_ was associated with a poor prognosis in patients with MINOCA. Routine assessment of HbA_1c_ enables an early recognition of pre-DM and thus may facilitate risk stratification in this specific population.

## Introduction

Patients with abnormal glucose metabolism have much worse outcomes than patients without after acute myocardial infarction (AMI), even in the setting of optimal medical therapy and revascularization with percutaneous coronary intervention (PCI) [1]. Among the glucometrics, glycated hemoglobin (HbA_1c_) reflects the average blood glucose level in the past 4 to 8 weeks and still serves as the golden standard to assess glycemic status [2]. Since 2013, the measurement of HbA_1c_ has been highly recommended by American Diabetes Association (ADA) to stratify glucose metabolism as follows: normoglycemia (NG, HbA_1c_ < 5.7%), prediabetes (pre-DM, 5.7% ≤ HbA_1c_ < 6.5%), and diabetes (DM, HbA_1c_ ≥ 6.5% or diagnosed DM) [3]. For the past decades, DM has been considered as a robust contributor to cardiovascular (CV) risks [4]. Further researches have shown that pre-DM is also an independent risk factor for occurrence of CV diseases (CVD) and is associated with an increased risk of adverse CV events [5].

Recently, a distinct group of patients with myocardial infarction with nonobstructive coronary arteries (MINOCA) has drawn an increasing awareness with the widespread use of coronary angiography in the management of AMI. It is reported that MINOCA accounts for 5–10% of all AMIs and disproportionately affects women and younger patients compared to those with AMI and obstructive coronary artery disease (CAD) [6–8]. The pathogenesis of MINOCA is varied and may include plaque rupture or erosion, vasospasm, embolism, dissection, microvascular dysfunction and/or supply/demand mismatch. Non-vascular diseases such as myocarditis or Takotsubo syndrome may also mimic the presentation of MINOCA [9]. Previous studies have found that patients with MINOCA may not necessarily have a benign prognosis and they are still at considerable risk of developing future events even under optimal medical treatments [10–14]. Therefore, it is necessary to find residual risk factors and explore their implications in MINOCA.

To date, few research has focused on the association between glucometabolic status and long-term prognosis following MINOCA, and the clinical significance of pre-DM defined by elevated HbA_1c_ in MINOCA patients remains largely unknown. Herein, we explored the impact of abnormal glucose metabolism on CV outcomes after MINOCA, and specifically, investigated the prognostic value of pre-DM in this distinct population.

## Methods

### Study population

**Fig. 1:**
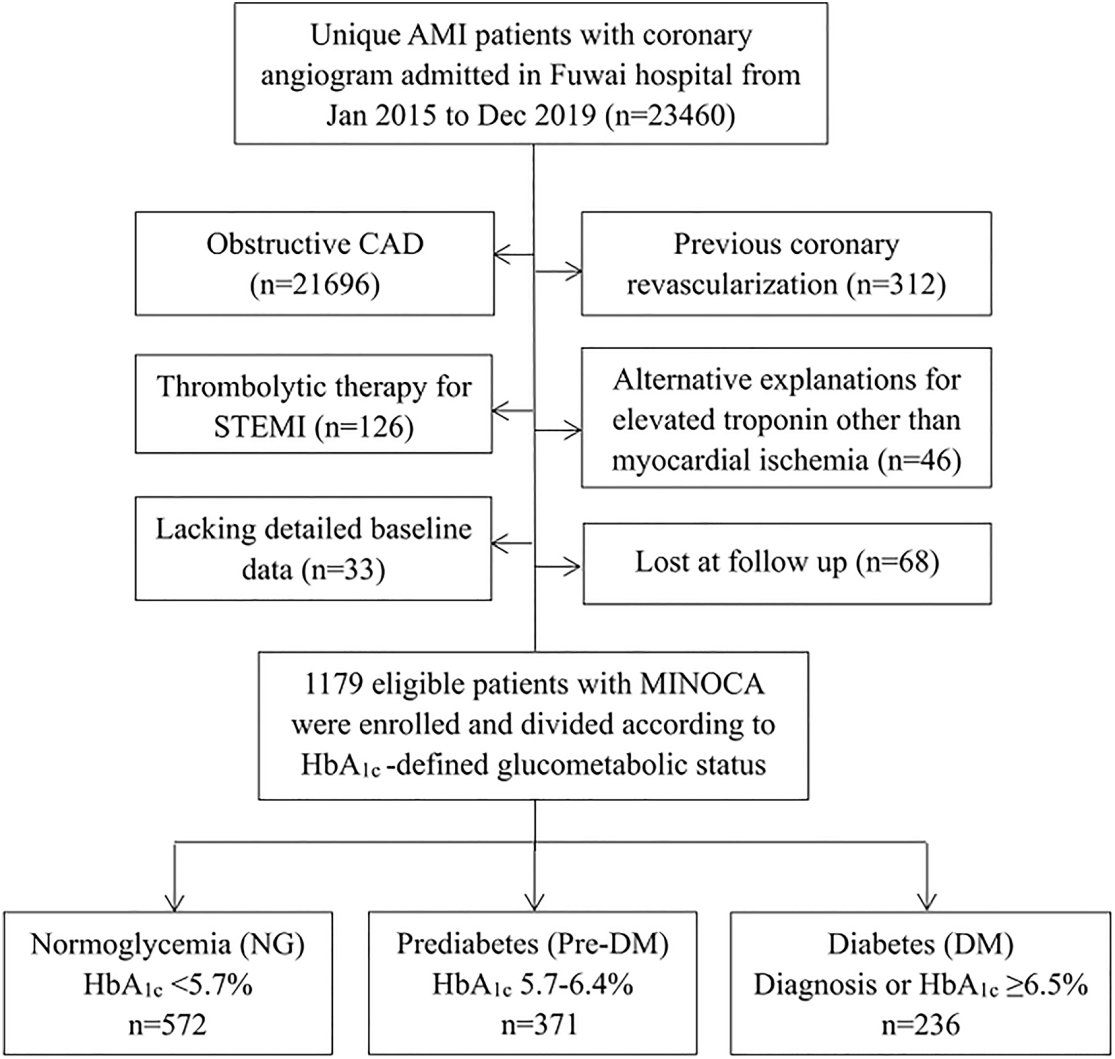
Study flowchart

This was a single-center, prospective and observational cohort study of patients with MINOCA. From January 2015 to December 2019, a total of 23,460 unique AMI patients with coronary angiogram were consecutively hospitalized in Fuwai hospital, including non-ST-segment elevation myocardial infarction (NSTEMI) and ST-segment elevation myocardial infarction (STEMI). Patients were diagnosed with MINOCA if they met the 4th universal definition of AMI [15] and the coronary angiography did not show a stenosis of ≥ 50% in epicardial coronary arteries [7]. Patients were excluded due to: (1) presence of obstructive CAD (n = 21,696); (2) prior revascularization (n = 312); (3) thrombolytic therapy for STEMI since the coronary lesion may be affected by thrombolysis (n = 126); (4) alternate explanations for elevated troponin rather than coronary-related causes (e.g., acute heart failure, myocarditis, pulmonary embolism, takotsubo syndrome, n = 46); (5) lack of detailed baseline data (n = 33); (6) lost at follow up (n = 68). Finally, 1179 eligible MINOCA patients were enrolled in this analysis (Fig. 1). All patients were prescribed the evidence-based optimal medical therapies during hospitalization, including dual anti-platelet therapy (DAPT), statins, angiotensin-converting enzyme inhibitor (ACEI) or angiotensin receptor antagonist (ARB), and β-blocker [16, 17]. This study was approved by the Ethics Committee of Fuwai hospital and complied with the Declaration of Helsinki. All enrolled subjects provided the written informed consent.

### Data collection

Patients’ demographics, medical history, laboratory test, echocardiographic data and medication were collected and verified from in-person interviews and medical records. Body mass index (BMI) was calculated as weight (kg) divided by height (m) squared. Glycated hemoglobin (HbA_1c_) was routinely measured at admission using an automated and high-performance liquid chromatography analyzer. Concentrations of fasting blood glucose (FBG), triglyceride (TG), total cholesterol (TC), low density lipoprotein cholesterol (LDL-C), high density lipoprotein cholesterol (HDL-C), creatinine, and high-sensitive C-reactive protein (hs-CRP) were tested with an automatic biochemistry analyzer. The N-terminal pro-B-type natriuretic peptide (NT-proBNP) and cardiac troponin I (TnI) values at admission were recorded. The left ventricular ejection fraction (LVEF) was measured by echocardiography using the biplane Simpson method.

### Definitions and outcomes

In the present study, glucometabolic status was defined in accordance with the ADA guideline [4]. DM was defined as having a history or newly diagnosed DM with HbA_1c_ ≥ 6.5%, FBG ≥ 7.0 mmol/L, or 2-h plasma glucose ≥ 11.1 mmol/L. Pre-DM was defined as 5.7% ≤ HbA_1c_ < 6.5% and HbA_1c_ < 5.7% indicated normoglycemia (NG). Notably, the pre-DM reflects the natural history of progression from NG to DM and represents those who have an impaired fasting glucose (IFG) or impaired glucose tolerance (IGT) [4]. Hypertension was defined as repeated blood pressure (BP) ≥ 140/90 mmHg, use of anti-hypertensive drugs, or having a history of hypertension. Dyslipidemia was defined by history or those with LDL-C ≥ 3.4 mmol/L, HDL-C < 1.0 mmol/L, or TG ≥ 1.7 mmol/L [18].

The primary study endpoint was a composite of major adverse cardiovascular events (MACE), including all-cause death, nonfatal MI, revascularization, nonfatal stroke, and hospitalization for unstable angina (UA) or heart failure (HF). The MACE was assessed as time to first event. The secondary endpoints included each component of MACE and the composite “hard” endpoint of death, nonfatal MI, revascularization, and nonfatal stroke. Reinfarction was diagnosed according to the 4th universal definition of MI [15]. Revascularization was performed at the operator’s discretion due to recurrent ischemia and progression of coronary lesion. Stroke was defined by the presence of neurological dysfunction and vascular brain injury induced by cerebral ischemia or hemorrhage [19]. Hospitalization for UA or HF reflected the clinical status and quality of life after AMI. Patients were regularly followed up at clinics or through telephone by the independent researchers. The endpoints were confirmed by at least two professional cardiologists.

### Statistical analysis

Data were expressed as mean ± standard deviation (SD) or median with interquartile range for continuous variables and numbers with percentages for categorical variables. Differences were evaluated using the analysis of variance or Kruskal–Wallis H test for continuous variables and Pearson’s χ^2^ or Fisher’s exact test for categorical variables. The cumulative incidence of adverse events among groups were showed by Kaplan–Meier analysis and compared using the log-rank test. The univariable and multivariable Cox regression analyses were used to identify relation between glucometabolic status and the event risk. The risk was adjusted by age and sex and further adjusted by multiple clinically relevant variables, including age, sex, BMI, MI classification (NSTEMI or STEMI), history of hypertension, and dyslipidemia. The hazard ratio (HR) with 95% confidence interval (CI) were calculated. A two-sided analysis with a P value < 0.05 was considered statistically significant. Data were analyzed using SPSS version 22.0 (SPSS Inc., Chicago, USA).

## Results

### Baseline characteristics

Patients were divided into NG, pre-DM and DM groups based on the HbA_1c_-defined glucometabolic status (NG, n = 572; pre-DM, n = 371; DM, n = 236) (Fig. 1). Compared with NG group, patients with pre-DM and DM were older and more often female (Table 1). Pre-DM and DM patients also had higher BMI, higher BP levels at admission, higher prevalence of hypertension and dyslipidemia, lower LVEF, higher NT-proBNP, higher values of TG, TC, LDL-C, and lower HDL-C level. Moreover, patients with pre-DM and DM were more likely to receive treatment with ACEI or ARB and β-blocker. The proportion of STEMI, and creatinine, hs-CRP, and TnI values were comparable among the 3 groups. In this regard, patients with abnormal glucose metabolism, especially DM, appeared to have more comorbidities and risk profiles at baseline.

**Table 1:** Baseline characteristics and clinical outcomes of MINOCA patients based on glucometabolic status

	Normoglycemia (n = 572)	Prediabetes (n = 371)	Diabetes (n = 236)	p value
Male, n(%)	437 (76.3%)	269 (72.5%)	161 (68.2%)	< 0.001
Age, yrs	53.0 ± 12.2	57.9 ± 9.8	58.2 ± 11.4	< 0.001
BMI, kg/m^2^	25.0 ± 3.6	25.5 ± 3.6	26.4 ± 4.2	0.007
STEMI, n(%)	235 (41.0%)	149 (40.1%)	91 (38.5%)	0.800
Emergent CAG, n(%)	79 (13.8%)	44 (11.8%)	36 (15.2%)	0.503
Vital signs at admission				
Systolic BP, mmHg	124.3 ± 17.5	124.4 ± 17.2	129.1 ± 17.7	0.001
Diastolic BP, mmHg	75.4 ± 11.0	76.4 ± 11.8	78.1 ± 12.5	0.022
Heart rate, bpm	69.1 ± 10.2	69.2 ± 11.7	70.5 ± 11.2	0.209
Medical history, n(%)				
Hypertension	272 (47.5%)	202 (54.4%)	156 (66.1%)	< 0.001
Dyslipidemia	316 (55.2%)	217 (58.4%)	153 (64.8%)	0.001
Previous MI	22 (3.8%)	21 (5.6%)	15 (6.3%)	0.252
Killip class ≥ 2, n(%)	38 (6.6%)	30 (8.0%)	21 (8.8%)	0.189
LVEF, %	61.1 ± 7.0	60.3 ± 7.6	59.3 ± 8.1	0.007
Laboratory data				
FBG, mmol/L	4.98 ± 0.53	5.40 ± 0.68	7.93 ± 2.59	< 0.001
HbA_1c_, %	5.40 ± 0.24	6.00 ± 0.19	7.38 ± 1.38	< 0.001
TG, mmol/L	1.39 (1.03, 1.95)	1.41 (1.05, 1.90)	1.70 (1.16, 2.33)	0.001
TC, mmol/L	3.83 ± 0.90	3.89 ± 0.84	4.12 ± 1.02	0.018
LDL-C, mmol/L	2.25 ± 0.63	2.28 ± 0.68	2.46 ± 0.85	0.033
HDL-C, mmol/L	1.11 ± 0.34	1.08 ± 0.29	1.04 ± 0.27	0.042
Creatinine, μmol/L	80.2 ± 17.3	79.3 ± 14.1	81.0 ± 23.5	0.484
NT-proBNP, pg/mL	312 (108, 652)	353 (117, 694)	453 (121, 786)	0.012
TnI, ng/mL	1.42 (0.32, 4.13)	1.47 (0.43, 3.92)	1.55 (0.54, 4.62)	0.113
hs-CRP, mg/L	2.12 (0.95, 5.50)	2.21 (1.07, 5.44)	2.22 (1.06, 7.12)	0.217
In-hospital medication				
DAPT	534 (92.8%)	342 (90.8%)	215 (93.8%)	0.515
Statin	544 (95.6%)	356 (94.6%)	230 (97.2%)	0.311
ACEI or ARB	347 (62.7%)	242 (63.4%)	170 (66.9%)	0.008
Beta-blocker	400 (71.1%)	276 (73.8%)	184 (73.7%)	0.049
CV outcomes				
MACE	62 (10.8%)	60 (16.1%)	46 (19.4%)	0.003
Death, nonfatal MI, stroke or revascularization	34 (5.9%)	37 (9.9%)	31 (13.1%)	0.005
All-cause death	6 (1.0%)	7 (1.8%)	5 (2.1%)	0.423
Nonfatal MI	16 (2.7%)	15 (4.0%)	10 (4.2%)	0.455
Revascularization	14 (2.4%)	16 (4.3%)	16 (6.7%)	0.014
Nonfatal stroke	3 (0.5%)	5 (1.3%)	4 (1.6%)	0.240
Hospitalization for UA	23 (4.0%)	26 (7.0%)	22 (9.3%)	0.010
Hospitalization for HF	19 (3.3%)	17 (4.5%)	12 (5.0%)	0.347

*BMI* body mass index, *STEMI* ST-segment elevation myocardial infarction, *CAG* coronary artery angiology, *BP* blood pressure, *LVEF* left ventricular ejection fraction, *FBG* fasting blood glucose, *HbA*_*1c*_ glycated hemoglobin, *TG* triglyceride, *TC* total cholesterol, *LDL*-*C* low-density lipoprotein cholesterol, *HDL-C* high-density lipoprotein cholesterol, *NT-proBNP* N-terminal pro-B-type natriuretic peptide, *TnI* troponin I, *hs-CRP* high-sensitive C-reactive protein, *DAPT* dual anti-platelet therapy, *ACEI* angiotensin-converting enzyme inhibitor, *ARB* angiotensin receptor antagonist, *MACE* major adverse cardiovascular events, *MI* myocardial infarction, *UA* unstable angina, *HF* heart failure

### CV outcomes

**Fig. 2:**
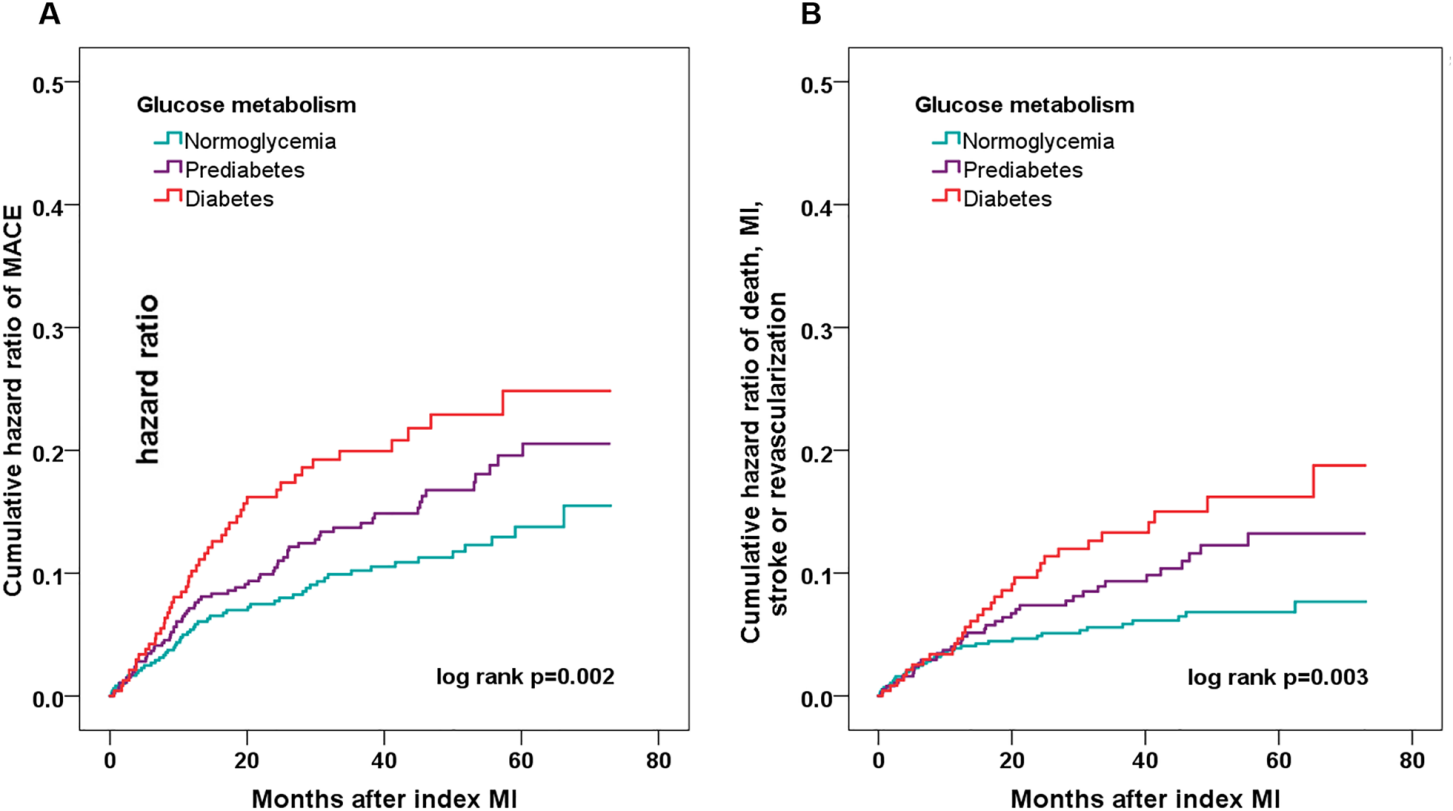
Kaplan–Meier analysis showing the cumulative hazard ratio of composite event in MINOCA patients stratified by glucometabolic status

Over the median follow-up time of 41.7 months, 168 MINOCA patients experienced MACE (18 died, 41 had reinfarction, 46 had revascularization, 12 suffered stroke, 71 was hospitalized for UAP and 48 hospitalized for HF) (Table 1). Patients with pre-DM and DM had a significantly higher incidence of MACE compared to NG group (10.8%, 16.1%, 19.4% for NG, pre-DM, and DM respectively; p = 0.003). The incidence of the composite endpoint of death, recurrent MI, stroke or revascularization also increased in pre-DM and DM groups (5.9%, 9.9%, 13.1%; p = 0.005). In addition, the Kaplan–Meier analysis indicated that the cumulative incidence of MACE and the composite endpoint were significantly higher in patients with pre-DM and DM (Fig. 2A, B).

### Association between glucometabolic status and outcomes

**Fig. 3:**
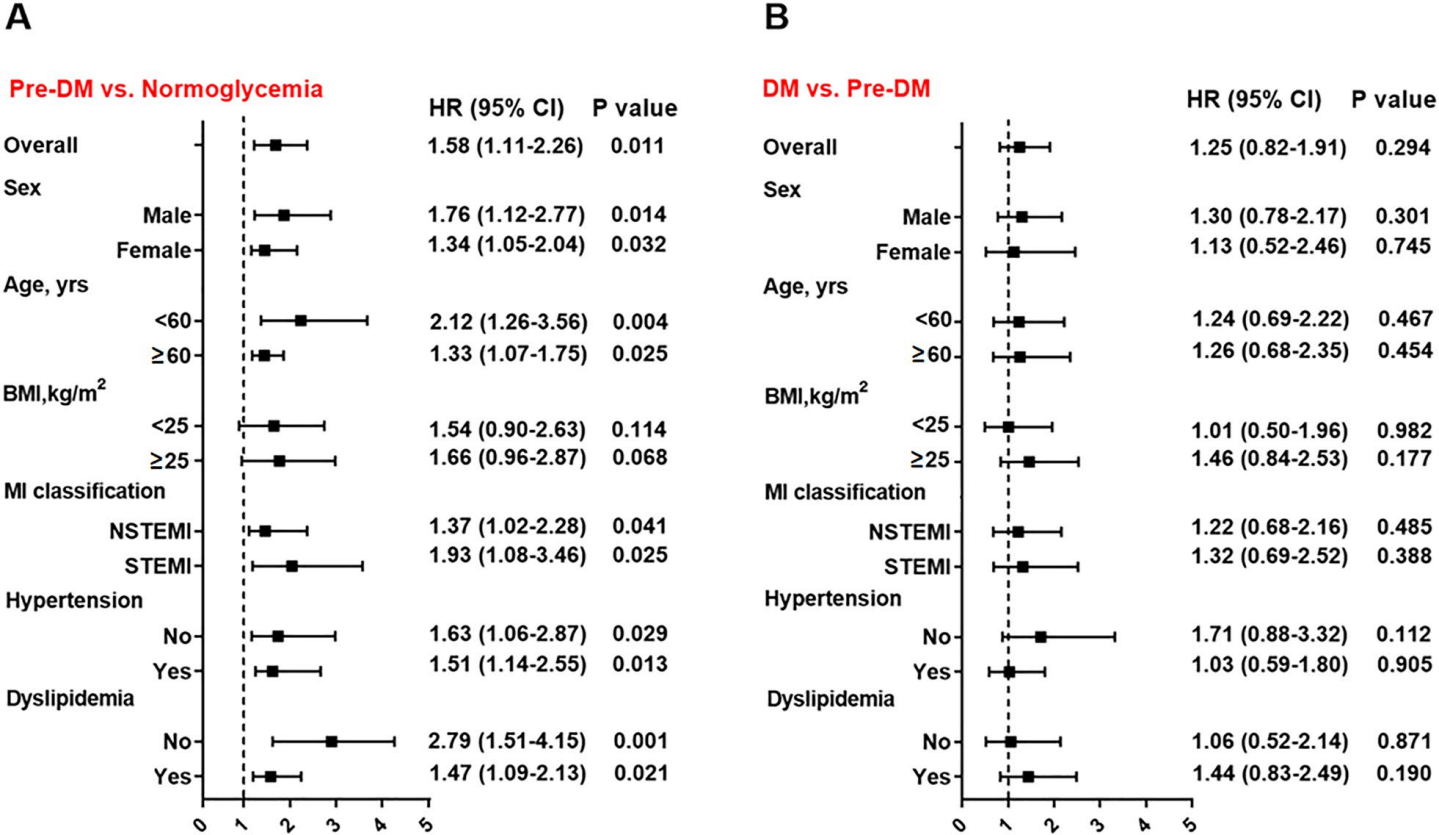
Impact of prediabetes on risk of MACE compared to normoglycemia or diabetes in overall and subgroups. Hazard ratio (HR) was calculated by univariate Cox regression analysis. Vertical dotted line indicated the HR value of 1. BMI: body mass index, STEMI: ST-segment elevation myocardial infarction, NSTEMI: non-ST-segment elevation myocardial infarction, DM: diabetes, pre-DM: prediabetes, CI: confidence interval

At multivariate Cox analysis, pre-DM was significantly associated with an increased risk of MACE after adjustment for age and sex (HR 1.49, 95% CI 1.07–2.11, p = 0.020) and after adjustment for multiple clinically relevant variables (HR 1.45, 95% CI 1.03–2.09, p = 0.042). As expected, DM was a robust risk factor of MACE after multivariate adjustment (HR 1.79, 95% CI 1.20–2.66, p = 0.005) (Table 2). The adjusted risk of the composite endpoint of death, recurrent MI, stroke or revascularization also markedly increased in patients with pre-DM and DM (all p < 0.05). At subgroup analysis, Pre-DM remained a risk factor of MACE compared with NG in subsets of patients stratified by sex, age, MI classification, history of hypertension, and dyslipidemia (expect for BMI) (Fig. 3A). Meanwhile, the risk of MACE was similar among patients with DM and pre-DM in overall and in subgroups (Fig. 3B), suggesting that pre-DM may potentially have a similar impact as DM on clinical outcomes in patients with MINOCA.

**Table 2:** Association between glucometabolic status and the event risk

Groups	Unadjusted		Adjusted Model 1		Adjusted Model 2	
	HR (95% CI)	P value	HR (95% CI)	P value	HR (95% CI)	P value
MACE						
Normoglycemia	Reference	…	Reference	…	Reference	…
Prediabetes	1.58 (1.11–2.26)	0.011	1.49 (1.07–2.11)	0.020	1.45 (1.03–2.09)	0.042
Diabetes	1.98 (1.35–2.90)	< 0.001	1.82 (1.23–2.70)	0.004	1.79 (1.20–2.66)	0.005
Death, MI, stroke or revascularization						
Normoglycemia	Reference	…	Reference	…	Reference	…
Prediabetes	1.72 (1.08–2.74)	0.022	1.69 (1.05–2.71)	0.028	1.67 (1.04–2.68)	0.033
Diabetes	2.35 (1.44–3.82)	< 0.001	2.09 (1.27–3.45)	0.002	2.04 (1.23–3.38)	0.004

*HR* hazard ratio, *CI* confidence interval, *MACE* major adverse cardiovascular events

Model 1 included age and sex. Model 2 included age, sex, BMI, MI type (NSTEMI or STEMI), hypertension, and dyslipidemia in multivariate Cox analysis

## Discussion

In the present study, we found that MINOCA patients with pre-DM had significantly higher event risks compared to those with NG, whereas the prognosis of pre-DM was comparable to DM patients. Our data, for the first time, verified the association between pre-DM and outcomes after MINOCA, highlighting its prognostic value and potential utility for risk stratification in the contemporary real-world management of MINOCA.

MINOCA is an interesting clinical entity with multiple potential causes. According to updated definitions [7], a working diagnosis of MINOCA should be only considered in patients with a definite AMI, nonobstructive coronary arteries based on angiography, and no other diseases that lead to myocardial injury without ischemia (e.g., myocarditis). We adopted this criteria and prospectively enrolled a genuine cohort of MINOCA with long-term follow-up. A systematic review estimated that the prevalence of MINOCA to be 6% in patients with AMI [8], which is close to the prevalence of 5.1% in our study. Compared to AMI and obstructive CAD, individuals with MINOCA are younger, more often female, have fewer comorbidities, and nearly two-thirds of them would present with NSTEMI [8]. Here, we described the clinical characteristics of MINOCA as well. Of note, we found that as many as 1.5% of MINOCA patients died and 14.2% of them had a MACE during the median follow-up of 3.5 years. Consistently, previous studies reported a considerably high risk of long-term mortality and CV events after MINOCA [10–14]. In some cohorts, patients with MINOCA even had a similar prognosis compared with MI-CAD [11–13]. These data suggest that the prognosis of MINOCA is not a trivial thing and may need more attention. Thus, there is a rationale to find contributors to this residual CV risk and further improve clinical outcomes for this population.

It has been widely acknowledged that patients with DM had a worse prognosis than those without after an AMI [1]. More importantly, pre-DM may also have an unfavorable effect on CV outcomes [5]. Although IFG or IGT defined by the oral glucose tolerance test (OGTT) is considered more sensitive than HbA_1c_ for defining abnormal glycemic status, a major advantage of measuring HbA_1c_ is that the HbA_1c_ can be detected at any time without fasting and is not affected by diet or stress [2], especially in an acute illness such as AMI. Further, the OGTT test was not routinely assessed for each patients in our cohort, thus we used the HbA_1c_ -based definition of pre-DM in line with previous studies and the IFG or IGT may not be discriminated. Actually, every 1% increase in HbA_1c_ is correlated with a higher risk of CV event with a relative risk of 1.07 [20]. Given its good accuracy in predicting incident DM and future CVD, an increasing number of research have taken HbA_1c_ as an alternative tool to diagnose pre-DM.

Till now, several studies have explored the relationship between pre-DM defined by HbA_1c_ and CV outcomes either in general populations or in different cohorts with CAD. An updated meta-analysis concluded that the community-dwellers with pre-DM had a significantly increased risk of composite CVD [5]. Among individuals without a history of CVD, pre-DM was independently associated with the subclinical myocardial damage and this damage accurately predicted future events [21]. Recent studies further revealed the predictive value of pre-DM in CAD patients. Kok et al. found that patients with pre-DM had significantly higher event risks than those with normoglycemia in all-comers treated with PCI [22]. Another Korean study confirmed that pre-DM showed a bad effect on outcomes in AMI patients who underwent PCI using new-generation drug-eluting stents [23]. Similarly, Wang et al. reported that patients with pre-DM had a poor prognosis compared to those with NG in a broad Chinese population requiring PCI [24]. However, some studies had different conclusions and found that pre-DM was not an independent predictor for CV events [25–27]. The reasons for the inconsistent results might be partially attributed to the differences of baseline risk profiles and follow-up time among different studies. If the comorbidities were comparable among patients with NG and pre-DM in a cohort with a limited follow-up period, the prognostic effect of pre-DM may not have emerged yet during a short observation. Still, given the positive relation between HbA_1c_ levels and the risk of incident DM and future CV events, the ADA has recommended a routine screening for pre-DM in asymptomatic adults [3]. Further, the 2019 European Society of Cardiology (ESC) guideline states that people with pre-DM should counsel about effective strategies to lower their elevated risk of CVD [4].

To our knowledge, there are no specific large-scale studies regarding the prognostic value of pre-DM in MINOCA population, a distinct entity who remain at high CV risks and should be paid more attention. China now has the world’s largest diabetes epidemic. A national cross-sectional survey reported that the estimated overall prevalence of DM was 10.9% and that of pre-DM was 35.7% among adults in China [28]. In our cohort, the prevalence of DM was 20.0% and that of pre-DM was as high as 31.4%. The baseline profiles differed among patients with different glucose metabolism. Compared to those with NG, the incidence and adjusted risk of MACE were significantly higher in patients with pre-DM and DM, suggesting that pre-DM was an independent predictor of MACE compared to NG. Furthermore, pre-DM had a similar impact as DM on long-term CV outcomes in this population. These data support the wisdom of taking pre-DM as a risk factor for CVD. In clinical practice, routine assessment of HbA_1c_ appears to be of value to identify subjects with increased event risk after MINOCA.

The potential mechanisms linking the pre-DM and worse outcomes are varied. Even borderline high HbA_1c_ levels may represent a state of poor glycemic control and insulin resistance. This long-term hyperglycemia can directly aggravate inflammation, trigger oxidative stress, exacerbate endothelial dysfunction, enhance foam cell formation, and promote smooth muscle proliferation [29–31]. All these pathophysiological changes can further induce atherosclerotic plaque formation in coronary arteries, hyper-coagulation state, and vascular remodeling, thereby leading to a poor prognosis after AMI [32]. Even pre-DM showed a bad impact on outcomes in our cohort, larger randomized controlled trials are still needed to confirm the causal relationship between pre-DM and prognosis following AMI. Also, the pathophysiological and therapeutic relevance of pre-DM in the management of AMI are far from elucidated and thus warrant further investigation.

## Limitation

Our study had several limitations. First, the patients were enrolled in a single center with a limited sample size, and the selection bias may exist. Hence, future nationwide or international studies of large MINOCA cohorts may be more representative. Second, given the observational design of our study, we did not use the multi-modality imaging approach to identify the exact causes for each patient. The effect of different etiologies on outcomes needs more research. Third, the OGTT was not routinely measured and we may not be able to further stratify patients with IGT or IFG. Whether HbA_1c_ can be used in place of OGTT for glucose status screening in Chinese population warrants to be further clarified. Fourth, the residual confounding cannot be fully excluded despite the multivariate adjustment and subgroup analyses, thus our findings should be verified in randomized clinical trials. Finally, we did not record the dynamic changes of HbA_1c_ levels, and its measurement during the follow-up may also be clinically significant.

## Conclusion

Patients with pre-DM had a poorer prognosis after MINOCA compared to those with normoglycemia, while the outcomes were similar among patients with pre-DM and DM. These data support the idea that pre-DM is a risk factor of CV events. In clinical practice, assessment of HbA_1c_ may help to identify pre-DM and further stratify high-risk patients presenting with MINOCA.

## Data Availability

The datasets used and/or analyzed during the current study are available from the corresponding author on reasonable request.

## Abbreviations

AMI: Acute myocardial infarction
CAD: Coronary artery disease
CI: Confidence interval
CV: Cardiovascular
DM: Diabetes
HbA_1c_: Glycated hemoglobin
HR: Hazard ratio
MACE: Major adverse cardiovascular events
MINOCA: Myocardial infarction with nonobstructive coronary arteries
NG: Normoglycemia
NSTEMI: Non-ST-segment elevation myocardial infarction
pre-DM: Prediabetes
STEMI: ST-segment elevation myocardial infarction
